# Impact of Vitamin D Therapy on the Progress COVID-19: Six Weeks Follow-Up Study of Vitamin D Deficient Elderly Diabetes Patients

**DOI:** 10.1177/20101058211041405

**Published:** 2021-09-01

**Authors:** Amin R. Soliman, Tarek Samy Abdelaziz, Ahmed Fathy

**Affiliations:** 1 Internal Medicine Department, 63527 Kasr al Ainy School of Medicine Cairo University, Egypt

**Keywords:** 1 alpha, 25-dihydroxyvitamin D3, 1,25-dihydroxyvitamin D3, SARS-CoV-2, COVID-19

## Abstract

**Background:**

Coronavirus disease-19 (COVID-19) is an ongoing pandemic causing considerable fatalities worldwide. Vitamin D modulates the immune response through effects on various cells, such as: macrophages, B and T lymphocytes, neutrophils, and dendritic cells.

**Aim:**

To explore whether supplementation of vitamin D, in the form of a single intramuscular cholecalciferol injection, to patients with diabetes, COVID-19, and low vitamin D levels could improve the prognosis of those patients.

**Methods:**

This was a placebo-controlled randomized prospective study. The study has two arms as follows: the intervention arm (40 vitamin D deficient diabetes elderly patients that acquired SARS-CoV-2), compared to the control arm (16 elderly diabetes patients, with deficient vitamin D with SARS-CoV-2). Patients in the intervention arm were given vitamin D as a single intramuscular injection (200,000 IU); patients in the control arm were given placebo. The primary outcome was mortality within 6 weeks of the diagnosis of COVID-19. Clinical, laboratory, treatment, and outcome data were recorded after 6 weeks of follow-up.

**Results:**

No significant difference in 6 weeks mortality was observed between patients who received vitamin D and patients who received placebo (17.5% vs 18.8%, *p* = 0.838). Age, presence of hypertension, and chronic obstructive pulmonary disease were independent predictors of mortality at 6 weeks.

**Conclusion:**

Vitamin D supplementation did not reduce the severity or mortality of COVID-19 at 6 weeks. Further large scale studies are required to explore the effect of vitamin D therapy on survival in patients with diabetes mellitus who acquire COVID-19.

## Introduction

Immune response to SARS-CoV-2 is complex and can lead to hyperactivation of both innate immunity and adaptive immunity (B and T lymphocytes). CD4 and CD8 T cells may be markedly reduced in COVID-19 infection according to several reports.^1^

Vitamin D has been shown to exert immune-modulator effect. Sufficient vitamin D is vital to maintain the function and harmony of T cell response and avoid autoimmunity.^2^ The active form (1α, 25-dihydroxyvitamin D_3_ [1,25(OH)_2_D_3_]) accelerates fetal lung maturity and augments the development of type-II pneumocytes.^3^ Type-II pneumocytes (the protective cells of the alveoli) are the primary target of corona-viruses, as ACE2 receptors are highly expressed on these cells.^4^ There is mounting evidence that vitamin D exerts important antiviral effects as well as immune-modulator effects. Vitamin D has been therefore proposed as adjuvant treatment in influenza and HIV.^5^ Vitamin D has a distinct protective role in adult respiratory distress syndrome (ARDS), through increased expression of ACE2 receptors and rennin production; with the effect of reducing lung permeability.^5^ Additionally, vitamin D counteracts excessive T helper cell type 1(Th1) response and thus intercepts the development of cytokine storm.^6,7^

Low level of vitamin D, in certain population, may increase susceptibility to infections (e.g., tuberculosis).^8^ Children with vitamin D deficiency have increased susceptibility to acute lower respiratory tract infections.^9^ Administration of high dose of vitamin D to mechanically ventilated patients has resulted in shorting of hospital stay in a small pilot study.^10^

New exciting evidence suggests that vitamin D deficient patients, who acquire COVID-19, may have significant increase in hospitalization, with sevenfold increase in mortality.^6^ Moreover, in a case report, phototherapy to correct vitamin D level has resulted in a mild clinical course in high risk COVID-19 patients.^6^

We hypothesize that supplementation of high dose of vitamin D3, to correct vitamin D deficiency in COVID-19 patients, might have a favorable effect on survival.

## Methods

### Study Design

This was a randomized placebo controlled prospective study of the effect of intramuscular injection of cholecalciferol on survival of COVID-19 patients. A cohort of 40 elderly diabetes patients that acquired SARS-CoV-2 in period between March 01,2020 and the end of May 2020 were recruited from a single general hospital in Cairo. Another 16 elderly diabetes age-matched patients having vitamin D deficiency were given a placebo (control arm). The local ethical committee of our hospital approved the study. All patients gave their personal consents.

Patients were followed-up for 6 weeks in our general hospital, and their clinical, laboratory, and outcome data were extracted from medical records.

Patients’ assessment included the following: full history and physical examination including BMI and abdominal perimetry. Laboratory data included vitamin D, urea, creatinine, sodium (Na), potassium (k), calcium (Ca), phosphate (P), chloride (Cl), and glycosylated hemoglobin (HbA1c) levels. Vitamin D was assessed using automated ELISA.

### Inclusion Criteria

Patients included in the study were elderly type 2 diabetes adults with age more than 60 years males and females having deficient serum vitamin D levels (less than 20 ng/mL).

All elderly vitamin D deficient diabetes patients were diagnosed with COVID-19 by positive throat-swab specimens for SARS-CoV-2 PCR.

Exclusion criteria were the following: a known history of renal stones, diagnosis of hypercalcemia within the past year, baseline serum total calcium level more than 10 mg/dl, established diagnosis associated with increased risk of hypercalcemia (e.g., metastatic cancer, sarcoidosis, multiple myeloma, and primary hyperparathyroidism), and cholecalciferol supplementation within last 6 weeks before recruitment. Other exclusion criteria include known malignancy, organ transplant, known chronic autoimmune diseases, and long-term systemic steroid use. Those patients were excluded from the study to avoid bias of other immunological factors.

### Randomization

Eligible patients who consented for the study were allocated computer-generated random numbers.

### Confirmation of SARS CoV-2 Infection

Diagnosis of COVID-19 was confirmed by polymerase chain reaction (PCR) using TaqPath RT-PCR COVID-19 Kit.

### Vitamin D Supplementation

Cholecalciferol and matched placebo (normal saline) were supplied in identical prefilled syringes from our local pharmacy, which was responsible for preparing syringes of both cholecalciferol and placebo. Vitamin D was scheduled to be given in a dose of 200.000 units intramuscularly once as a single dose during the period of the study.

The 6 weeks morbidity and mortality were confirmed by reviewing individual medical and hospital discharge reports.

### Statistical Methods

Data were coded and entered using the statistical package for the Social Sciences version 26 (IBM Corp. Armonk, NY, USA). Data were summarized using mean and standard deviation for quantitative variables and frequencies (number of cases) and relative frequencies (percentages) for categorical variables. Comparisons between groups were done using unpaired t test when comparing two groups and analysis of variance (ANOVA) with multiple comparisons post hoc test when comparing more than two groups For comparing categorical data, Chi square test was performed. Fisher-exact test was used instead when the expected frequency is less than 5. *p*-values less than 0.05 were considered as statistically significant.

## Results

There were no statistically significant differences between the intervention arm and the placebo as regard epidemiological data (Table 1). Prevalence of comorbidities is shown in (Table 2). There was no significant difference in mortality (17.8% in the intervention arm vs 18.8% in the control arm, *p* = 0.83). There was no significant difference in the number of patients that needed intubation as shown in (Table 3). Logistic regression was performed to identify predictors of mortality in patients with confirmed COVID-19 infection (Table 4). Age, presence of hypertension, and COPD were independent predictors of mortality. Neither vitamin D level nor vitamin D therapy was significantly associated with mortality.

**Table 1.** Baseline patients charecteristics of vitamin D treated patients versus placebo treated.

	Vitamin D treated		Placebo treated		*p* value
	Mean	Standard deviation	Mean	Standard deviation	
Age (years)	71.30	4.16	70.19	4.57	NS
BMI	29.29	2.67	29.83	2.19	NS
Abdominal perimetry	106.37	7.32	110.94	8.51	NS
Hemoglobin (g/l)	137.90	3.10	136.94	2.24	NS
Total white cell count	4.32	0.33	4.21	0.27	NS
Lymphocytes	1.97 × 10^9^/L	0.54	1.93 × 10^9^/L	0.63	NS
Platelet count	263 × 10^9^/L	32	255 × 10^s^/L	29	NS
Aspartate transaminase	25.4 IU/L	9.5	24.9 IU/L	8.7	NS
Alanine transaminase	28.7 IU/L	11.03	27.9	9.17 IU/L	NS
Total bilirubin	1.71 mg/dl	0.6	1.65 mg/dl	0.61	NS
Direct bilirubin	0.5 mg/dl	0.2	0.45 mg/dl	0.17	NS
LDH	226.9 IU/L	18.6	233.4	17.1	NS
D-dimer	581 mg/dl	28.2	591	30.2	NS
ESR	34	8	36	11	NS
CRP	22 mg/dl	5.1	24.2	7.2	NS
Oxygen saturation (SPO2)	93	4	92	5	NS
Chloride	104.55	1.95	103.50	1.86	NS
C ( mmol/L)	2.35	0.13	2.28	0.12	NS
Urea (mg/dl)	40.83	3.71	41.56	3.98	NS
Creatinine (mg/dl)	1.31	0.17	1.29	0.15	NS
P (mmol/L)	0.96	0.07	0.98	0.05	NS
HbA1C (%)	7.21	0.38	7.47	0.32	NS
Vitamin D (ng/ml) (1st measurement)	10.4	1.3	21.17	3.96	0.001
Vitamin D (ng/ml) (2nd measurement)	20.54	3.00	21.23	3.98	NS
CT finding bilateral GGO no (%)	(19) 47%		(7) 45%		NS
CT finding crazy paving no. (%)	(7) 17%		(3) 20%		NS

**Table 2.** Co-morbidities in both groups.

Comorbidity	Vitamin D treated (40 patients	Placebo treated (16 patients)	*p* value
Hypertension	18 (45%)	7 (44%)	0.325
Cardiovascular disease	9 (23%)	4 (25%)	0.117
Chronic obstructive pulmonary disease	15 (38%)	8 (40%)	0.09
Malignancy	3 (8%)	1 (6%)	0.07
Chronic kidney disease	6 (15%)	3 (19%)	0.06

**Table 3.** Outcomes in the Intervention arm  versus the control arm.

		Vitamin D treated		Placebo treated		*p* value
		Count	%	Count	%	
Outcome	Death	7	17.5%	3	18.8%	0.838
	Intubation	14	35%	7	25.0%	
	Recovery	26	65.0%	9	56.3%	
Outcome	Death or intubation	14	35.0%	7	43.8%	0.541
	Recovery	26	65.0%	9	56.3%

**Table 4.** Logistic regression for the predictors of mortality of COVID-19 patients.

	Sig	Odds ratio	95% C.I. for odd ratio	
			Lower	Upper
Vitamin D level	.152	.797	.635	1.062
Age	.004	2.118	1.310	4.363
Vitamin D therapy	.213	.319	.0.71	4.721
Female sex	.068	8.436	.721	56.421
COPD	0.03	1.811	1.253	2.456
Hypertension	0.02	1.531	1.12	1.92

## Discussion

The relation between vitamin D status and the severity of COVID-19 is controversial. It is not known whether restoring vitamin D to normal levels would provide a potential treatment for COVID-19. Epidemiological studies have reported that insufficient vitamin D is associated with developing viral respiratory tract infections and acute lung injury. Vitamin D may have multiple biological and immunological roles to prevent the development of acute lung injury. One suggested mechanism is the inhibitory effect of vitamin D on the expression of members of the renin–angiotensin system such as ACE2 in lung tissue. Theoretically, vitamin D deficiency may act as a pathogenic factor in COVID-19. A recent meta-analysis of observational studies showed significantly greater risk of acute respiratory tract infection (ARTI) in patients with low levels of vitamin D.^11^ Evidence is accumulating that vitamin D supplementation may prevent the development of ARTI.^12^

Our randomized-controlled pilot study tested whether bolus therapy with vitamin D, based on vitamin D deficiency in patients with diabetes that acquired COVID-19, would provide any morbidity or mortality advantage. We treated patients with a large single dose of vitamin D rather than daily low dose, aiming for rapid correction of vitamin D status. Bolus therapy has been shown in previous studies to be more effective than daily low dose in achieving sufficient vitamin D level.^13^ Our main finding was that bolus large dose vitamin D therapy to correct vitamin D deficiency has not resulted in any benefit on the outcome measures (intubation, death, or recovery). There was a significant correlation between the outcome of our three groups (death, intubation, and recovery) and the age (*p* value 0.021). Moreover, age was independent predictor of worse clinical outcome (death or intubation, *p* = 0.047).

However, baseline vitamin D level was not independently associated with the outcome (death, intubation, or recovery).

The correlation between vitamin D and COVID-19 has gained special attention. In one large retrospective study, no correlation between low levels of vitamin D and the risk of acquiring COVID-19 infection was found. The study included biobank samples from 348,598 participants in the UK Of them, 449 had confirmed COVID-19 infection. Therefore, the main limitation of this study was that vitamin D level recording was long time before the advent of SARS-CoV-2 pandemic which signals a potential bias.^14^

On the other hand, many studies have found that vitamin D deficiency is an important determinant of the clinical course and outcome in patients with COVID-19. A systematic review reported low level of vitamin D in elderly people in Spain and Italy, the countries with very severe COVID-19 affection. This may interestingly suggest the potential relationship between low vitamin D level and risk of developing COVID-19.^15^ In a retrospective analysis, patients in Switzerland that acquired COVID-19 had significantly low levels of vitamin D.^16^ In spite of this obvious causal relation, few interventional studies failed to show clinical benefit of bolus vitamin D supplementation on prognosis of COVID-19 patients; in one randomized-controlled trial, vitamin D3 supplementation as single oral dose of 200.000 IU did not improve length of hospital stay or mortality outcome in COVID-19 patients.^17^ According to the National Institute for Health and Care Excellence (NICE) rapid evidence review, no robust evidence was found to support using vitamin D supplements to reduce the risk or severity of SARS-CoV-19.^18^

Our study has some limitations; first, the study duration was short which did not allow to observe the late outcomes. Second, only a small number of patients were recruited to the study.

## Conclusion

Vitamin D supplementation did not reduce the severity or mortality of COVID-19 at 6 weeks. Further large scale studies are required to explore the effect of vitamin D therapy on survival in patients with diabetes mellitus who acquire COVID-19.
